# Influence of Cabbage (*Brassica oleracea*) Accession and Growing Conditions on Myrosinase Activity, Glucosinolates and Their Hydrolysis Products

**DOI:** 10.3390/foods10122903

**Published:** 2021-11-23

**Authors:** Omobolanle O. Oloyede, Carol Wagstaff, Lisa Methven

**Affiliations:** Department of Food and Nutritional Sciences, Harry Nursten Building, University of Reading, Whiteknights, Reading RG6 6DZ, UK; c.wagstaff@reading.ac.uk (C.W.); l.methven@reading.ac.uk (L.M.)

**Keywords:** *Brassica oleracea*, cabbage, growing condition, myrosinase activity, glucosinolates, glucosinolate hydrolysis products, isothiocyanates, nitriles, epithionitriles

## Abstract

Glucosinolates are secondary plant metabolites present in *Brassica* vegetables. The endogenous enzyme myrosinase is responsible for the hydrolysis of glucosinolates, yielding a variety of compounds, including health-promoting isothiocyanates. The influence of cabbage accession and growing conditions on myrosinase activity, glucosinolates (GSL) and their hydrolysis products (GHPs) of 18 gene-bank cabbage accessions was studied. Growing conditions, cabbage morphotype and accession all significantly affected myrosinase activity and concentration of glucosinolates and their hydrolysis products. In general, cabbages grown in the field with lower growth temperatures had significantly higher myrosinase activity than glasshouse samples. Profile and concentration of glucosinolates and their hydrolysis products differed across the accessions studied. Aliphatic glucosinolates accounted for more than 60 % of total glucosinolates in most of the samples assessed. Nitriles and epithionitriles were the most abundant hydrolysis products formed. The results obtained showed that consumption of raw cabbages might reduce the amount of beneficial hydrolysis products available to the consumer, as more nitriles were produced from hydrolysis compared to beneficial isothiocyanates. However, red and white cabbages contained high concentrations of glucoraphanin and its isothiocyanate, sulforaphane. This implies that careful selection of accessions with ample concentrations of certain glucosinolates can improve the health benefits derived from raw cabbage consumption.

## 1. Introduction

Cabbage (*Brassica oleracea*) belongs to the *Brassicaceae* family and comprises eight distinct cultivar groups, all descended from wild cabbage (*B. oleracea* var. *oleracea*) [1]. Epidemiological studies have shown that the consumption of *Brassica* vegetables reduces the risks of cardiovascular diseases and cancer [2] and is reported to have a cytoprotective effect against tissue damage associated with oxidative stress as well as antimicrobial activity against bacterial and fungal pathogens [3,4].

*Brassica* vegetables are unique in comparison to other vegetables because they contain the enzyme myrosinase and a group of thioglucosides called glucosinolates (GSLs). GSLs are sulphur and nitrogen containing biologically active secondary metabolites found in plants of the order Capparales, which includes the *Brassicaceae* family and other economically important agricultural crops [5,6,7]. In plants, GSLs act as plant defense mechanisms against stress, insect, and pest attack [8]. GSLs have been grouped into three main classes based on the structure of their different amino acid precursors; these groups are aliphatic, aromatic and indole GSLs. Aliphatic GSLs are derived from alanine, leucine, isoleucine, methionine or valine; aromatic GSLs are from phenylalanine or tyrosine, while tryptophan-derived GSLs are called indole GSLs [9,10]. A recent review by Blažević et al. [11] stated that between 88–137 glucosinolates (GSLs) have been characterised in plants to date.

GSLs and myrosinase enzymes coexist in separate compartments in the plants; while glucosinolates exists in the vacuoles of various cells [6], myrosinase enzymes are localised inside the myrosin cells. When plant tissue is disrupted, GSLs are hydrolysed by plant myrosinase enzymes, resulting in the formation of various hydrolysis products such as isothiocyanates (ITCs), thiocyanates, nitriles and epithionitriles [5]. The extent of glucosinolate hydrolysis and the type of hydrolysis compound produced is dependent on a number of factors, which include coexisting myrosinase enzyme, presence of epithiospecifier protein (ESP), ascorbic acid, Fe^2+^ and MgCl_2,_ structure of the glucosinolate side chain, the plant species, as well as reaction conditions such as pH and temperature [9,12,13].

ITCs, the primary products of GSL hydrolysis from myrosinase, are responsible for the well-documented health-promoting properties of *Brassica* vegetables, such as reduced risk of cardiovascular diseases (CVD) and cancer [2,5]. For example, sulforaphane (SFP), the hydrolysis product of glucoraphanin present in high concentrations in broccoli and cabbage, has been reported to possess chemoprotective, antimicrobial, anti-inflammatory, and antithrombotic properties [14,15]. Allyl isothiocyanate (AITC), another common ITC present in cabbages and produced upon myrosinase hydrolyses of the glucosinolate sinigrin (SIN), was reported to be potent against human breast cancer cells [16], human erythroleukemic K562 cells [17], and more potent on human A549 and H1299 non-small cell lung cancer cells in vitro than 2-phenylethyl-ITC (PEITC; ITC from gluconasturtin) [18]. However, in the presence of epithiospecifier proteins (ESPs), nitriles and epithionitriles (EPTs), which have not been shown to proffer any beneficial characteristics for health, are formed [19]. GSLs and ITCs are also partly responsible for the bitter taste and pungent aromas of *Brassica* vegetables, which limits consumer acceptance and liking of *Brassica* vegetables [20,21,22,23].

There are several factors that affect the GSL-myrosinase system in *Brassicas*; these factors include climatic factors, location, and growing conditions [24,25,26,27], morphotype and the variety of plant [28,29]; with the impact of these factors varying between studies. For example, while some authors have suggested that the effect of plant genotype on GSL concentrations is greater than that of environmental factors [30,31], others have reported higher variations in GSL concentrations as a result of environmental conditions than genetic factors [32,33].

To date, most studies on myrosinase activity have focused on single cultivars of B. *oleracea* species [28,34,35,36,37], with studies on the myrosinase activity of different varieties within a species limited [29,38]. Variations in myrosinase activities were reported in different varieties of Brussels sprouts, broccoli, cauliflower, Chinese cabbage, and white cabbage [38]. The authors found a two-fold difference in the myrosinase activities of five broccoli varieties as well as two cauliflower and Chinese cabbage varieties. Low temperature conditions are reported to increase the myrosinase activity of *B.* *oleracea* species (Brussels sprout, broccoli, cauliflower, cabbage, and kale) grown in the autumn season [25].

Several studies have been undertaken on the formation of GSLs in cabbage varieties, some of which have investigated GSL concentrations in cabbages grown under different conditions; with , most focused on GSL concentrations of cabbages grown in different locations or different seasons [29,32,39,40,41]. However, none of the studies analysed the glucosinolate hydrolysis products (GHPs) of cabbages under different plant growth conditions and instead made suggestions on potential GHP concentrations of the samples based on the concentrations of GSLs observed. These suggestions may be problematic, as studies have shown that GSL concentration is not necessarily correlated with the abundance of GHPs formed [42,43].

Little is known of the GHPs in cabbages, as most studies have focused on a specific cabbage variety [44] or ITCs in other *B. oleracea* such as broccoli [45]. A recent study analysed the GSLs and GHPs of cabbages with a focus on red, white and savoy cabbages, but the samples were grown under the same conditions [43]. To fully understand the health benefits that can be derived from cabbage consumption, however, there is a need to characterize the GHPs produced from GSL hydrolysis and understand the factors affecting the type and concentrations of GHPs formed. With growing health campaigns promoting the consumption of more fruits and vegetables, and consumers wanting to include more fresh vegetables like cabbage in their diet, many people now grow their own cabbages at home in pots, either in green/glasshouses or in the garden [46,47]. It is therefore important to investigate the effect of these plant growth conditions on the GSL-myrosinase system to ensure that the health benefits desired from their consumption are not lost.

In light of this gap in present knowledge, the purpose of this study was to investigate the influence of growing conditions and accession identity on myrosinase activity as well as the GSL and GHP content of cabbage. A total of 18 cabbage accessions across six different cabbage morphotypes were selected from a genetic resources unit and grown under two different conditions. In addition to wild cabbage, this study used red, white, and green (savoy) cabbage (*B. oleracea* var. *capitata*), kale (*B. oleracea* var. *acephala*) and sea kale (*B. oleracea* var. *tronchuda*). The primary hypothesis of the study was that cabbage growth conditions will affect myrosinase activity as well as the GSL and GHP contents of cabbage. The secondary hypothesis was that while cabbage morphotype and accession would affect myrosinase activity, cabbage morphotype rather than accession will affect the profile and concentrations of the GSLs and GHPs formed. The results of myrosinase activity and variations in the amount and profile of GSLs and GHPs in cabbage accessions across both plant conditions studied are presented.

## 2. Materials and Methods

### 2.1. Plant Material

Cabbage accessions were selected from the University of Warwick Crop Centre Genetic Resources Unit (Wellesbourne, UK). Eighteen cabbage accessions comprising six cabbage morphotypes (wild (*B. oleracea* var. *oleracea*), black kale (*B. oleracea* var. *acephala*), tronchuda (*B. oleracea* var. *tronchuda*), savoy, red and white (*B. oleracea* var. *capitata*)) were used for the experiment. Cabbages were selected based on their geographical origin, whether or not they were of hybrid descent, and morphology of head formation (closed heart or open leaf), as shown in Table 1 and Supplementary Figure S1. Seeds of one white cabbage accession (WC-DLI) did not germinate when sown and thus will not be discussed further. Out of the remaining 17 accessions planted, RC-RM (red cabbage) and SC-SDG (savoy cabbage) did not survive in the glasshouse.

A total of 15 biological replicates of each accession were germinated in seedling trays using potting compost under controlled environmental conditions (Saxcil cabinets). A 16 h photo period was used (16 h light, 8 h dark); relative humidity was set to 60%, with day and night temperatures of 22 °C and 16 °C, respectively. Seedlings were allowed to grow in seedling trays until the appearance of 3–4 true leaves, before being transplanted to individual 2.5 L pots containing loam-based compost (7–8 May 2014) and left to grow in the glasshouse (minimum night temperature 13 °C). After 50 days (26–27 June 2014), five replicates of each accession were transplanted to larger pots (10 L) containing loam-based compost and allowed to grow until commercial maturity in the glasshouse, while seven replicates of each accession were transplanted to the field and allowed to grow to commercial maturity. On the field, each accession was planted on 7 metre beds with 0.6 metres between plants and rows. Both glasshouse and field cabbages were fertilized twice weekly with nitrogen phosphate potassium (NPK) (100 kg/ha N, 100 kg/ha P and 200 kg/ha K) fertilizer. Standard agricultural practices were employed in the cultivation of the cabbages, including a programme of pest management using insecticides and fungicides. Cabbages were grown between 7 March–25 November 2014 in the plant growth facilities, Whiteknights campus of the University of Reading, UK (Supplementary Figure S2).

Cabbages were harvested over a period of two days upon reaching commercial maturity based on visual inspection. Though some accessions attained commercial maturity earlier than others, they had sufficiently good field holding capacity to be left until all accessions were mature before harvesting, so that all plants experienced equivalent environmental conditions. Harvested plants were placed on ice in freezer bags and immediately stored in a cold room at 4 °C for 24 h before processing. The average weight of each field cabbage head per plant was 700 g (closed heart) and 300 g (open leaf), while the glasshouse cabbages were smaller (400 g for closed heart and 250 g for open leaf cabbages) (Supplementary Figure S1). Climatic data for both growing conditions are presented in Supplementary Table S1.

### 2.2. Reagents and Chemicals

Sinigrin standard was purchased from Santa Cruz Biotechnology (Heidelberg, Germany) and D-glucose determination kit was from R-Biopharm Rhone (Heidelberg, Germany). All other chemicals used were purchased from Sigma–Aldrich (Dorset, UK).

### 2.3. Sample Preparation

The outer leaves and central core of 4–5 cabbage heads (biological replicates) were removed and discarded in order to remove senescent leaves and achieve a representative sample spanning similar leaf ages for each morphotype. Cabbages were chopped into pieces of approximately 1 cm in width using a kitchen knife (representing how cabbages would normally be sliced by consumers), mixed together, and washed under running tap water; excess water was drained using a salad spinner (OXO Good Grips Clear Manual Salad Spinner, Chambersburg, PA, USA). A total of 120 g of cabbage samples was put into sterile sterilin tubes, immediately placed on ice, and transferred to a −80 °C freezer. Frozen samples were freeze-dried (Stokes freeze drier, Philadelphia, PA, USA), ground using a tissue grinder (Thomas Wiley^®^ Mini-Mill, Thomas Scientific, Swedesboro, NJ, USA) and stored at −20 °C until further analysis.

### 2.4. Myrosinase Enzyme Extraction and Assay

Myrosinase enzyme was extracted using the method described by Ghawi et al. [48]. A sample of 0.1 g was suspended in 0.15 g polyvinylpolypyrrolidone (PVPP) and 10 mL of Tris-HCL buffer (200 mM, pH 7.5) containing 0.5 mM ethylenediaminetetracetic acid (EDTA) and 1.5 mM dithiothreitol (DTT). The mixture was stirred for 15 min at 5 °C and centrifuged (11,738× *g*) for 15 min at 5 °C. The final volume of supernatant was made up to 10 mL using the Tris-HCL buffer. Then, 6.2 g ammonium sulphate was added to the supernatant to achieve 90% saturation and stirred at 5 °C for 30 min. The samples were then centrifuged (13,694× *g*) for 15 min at 5 °C. The resulting pellet was suspended in 2 mL Tris-HCl buffer (10 mM, pH 7.5) and assayed for myrosinase activity.

Myrosinase activity was measured using the coupled enzyme method described by Gatfield and Sand [49] and Wilkinson et al. [50], with slight modifications. The procedure depends on the glucose released from the reaction between myrosinase enzymes and the substrate (sinigrin). The mixture for the reaction consisted of 0.9 mL of 5 mM ascorbic acid, 0.5 mL ATP/NADP+ solution, 10 µL hexokinase/glucose-6-phosphate dehydrogenase and 50 µL crude enzyme extract. The mixture was homogenized and allowed to stand for 3 min, and then 50 µL sinigrin substrate (0.6 M) added. The change in absorbance due to NADPH formation was read on a spectrophotometer at 340 nm. Myrosinase enzyme activity was determined by taking the slope of the linear part of the curve of absorbance versus the time of reaction. One unit of myrosinase activity is defined as the amount of enzyme that produces 1 µmol of glucose from sinigrin substrate per minute at pH 7.5.

### 2.5. Protein Assay

Protein content was measured using the Bradford method [51]. The procedure is based on formation of a complex between dye (brilliant Blue G, Sigma-Aldrich, Dorset, UK) and the protein present in the sample, and absorbance is read at 595 nm using a spectrophotometer. 50 µL filtered crude enzyme extract was added to 1.5 mL of concentrated dye reagent, vortexed and allowed to stand for 20 min before taking the absorbance reading. Bovine serum albumin (BSA) (Sigma-Aldrich, Dorset, UK) was used to construct a standard curve, and the protein concentration of sample was calculated from the standard curve obtained. Protein content was used to calculate the specific activity of myrosinase enzymes (U/mg protein).

### 2.6. Glucosinolate Extraction and LC-MS^2^ Analysis

The method used for GSL extraction is as described by Bell et al. [52], with modifications. Briefly, 40 mg ground cabbage powder was heated in a heat block at 75 °C for two minutes. Then, 1 mL 70% (*v*/*v*) methanol preheated to 70 °C was added to each sample, vortexed and placed in a preheated (70 °C) water bath for 20 min. Samples were centrifuged at full speed for five minutes (18 °C), and supernatant was collected in fresh Eppendorf tubes. The volume was adjusted to 1 mL with 70% (*v*/*v*) methanol and frozen at −80 °C until further analysis.

Samples were filtered using 0.22 μm Millex syringe filters with a low protein binding Durapore polyvinylidene fluoride (PVDF) membrane (Fisher scientific, Loughborough, UK) and diluted with 9 mL HPLC-grade water. LC-MS analysis of GSL extracts was performed in negative ion mode on an Agilent 1200 Series LC system (Agilent, Stockport, UK) equipped with a variable wavelength detector and coupled to a Bruker HCT ion trap (Bruker, Coventry, UK). Sample separation was achieved on a Gemini 3 μm C_18_ 110 Å (150 × 4.6 mm) column (with Security Guard column, C_18_; 4 mm × 3 mm; Phenomenex, Macclesfield, UK). GSLs were separated during a 40 min chromatographic run, with a 5 min post-run sequence. Mobile phases consisted of 95% of 0.1% ammonium formate solution and 5% acetonitrile. The flow rate was optimised for the system at 0.4 mL/min, with a column temperature of 30 °C and with 5 μl of sample injected into the system. GSLs were quantified at a wavelength of 229 nm.

MS analysis settings were as follows: electrospray ionization (ESI) was carried out at atmospheric pressure in negative ion mode (scan range m/z 100–1500 Da). Nebulizer pressure was set at 50 psi, gas-drying temperature at 350 °C, and capillary voltage at 2000 V. GSLs were quantified using sinigrin hydrate standard. Five concentrations of sinigrin hydrate (14–438 µg/mL) were prepared with 70% methanol and used to prepare an external calibration curve (*r*^2^ = 0.996). Compounds were identified using their parent mass ion and characteristic ion fragments as well as comparing with literature ion data (Table 2). Compounds were quantified using Bruker Daltonics HyStar software (Bruker, Coventry, UK). Relative response factors (RRFs) were used in the calculation of GSL concentrations where available [53]. Where such data could not be found for intact GSLs, RRFs were assumed to be 1.0.

### 2.7. Extraction of Glucosinolate Hydrolysis Products

GHPs were extracted and analysed following the method described by Bell et al. [57]. A total of 0.5 g of lyophilized cabbage was mixed with 10 mL deionized water, vortexed and allowed to incubate for three hours at 30 °C. The mixture was then centrifuged at 5000× *g* (18 °C) for ten minutes, and the supernatant collected. The pellet was extracted two more times with 10 mL deionized water, and the supernatants were combined and filtered (0.45 μm syringe filters, Epsom, UK) into glass centrifuge tubes. GHPs were extracted by adding an equal volume of dichloromethane (DCM) to the supernatant, vortexed for one minute and centrifuged at 3000× *g* for ten minutes. After centrifugation, the organic phase was collected, and the extraction step repeated twice. The organic phase collected was combined, 2 g sodium sulphate salt was added to remove any excess liquid present, and the mixture was filtered into a round-bottom flask. The filtrate was dried using a rotatory evaporator (37 °C), re-dissolved in 1 mL DCM, and filtered (0.22 μm filter; Fisher scientific, Loughborough, UK) in GC-MS glass vials (VWR, Lutterworth, UK) for GC-MC analysis.

### 2.8. GC-MS Analysis

GC–MS analysis was performed on an Agilent 7693/5975 GC–MS autosampler system (Agilent, Manchester, UK). The sample was injected onto a HP-5MS 15 m non-polar column DB-5MS (J and W scientific, Santa Clara, CA, USA) (0.25-μm film thickness, 0.25 mm I.D.). The injection temperature was 250 °C in split mode (1:20). The oven temperature was programmed from 40 to 320 °C at a rate of 5 °C/min until 250 °C. The carrier gas was helium, with flow rate of 1.1 mL/min and pressure of 7.1 psi. Mass spectra were obtained by electron ionization at 70 eV, and mass scan from 35 to 500 amu. A total of 1 μL of the sample was injected, and compounds were separated during a 42 min run. Compounds were identified using the National Institute of Standards and Technology (NIST) library and literature ion data (Table 3; see Figure S3 for GC-MS chromatograms) and quantified based on an external standard calibration curve. Five concentrations (0.15–0.5 mg/mL) of sulforaphane standard (Sigma Aldrich, Dorset, UK) were prepared in DCM (*r*^2^ = 0.99). Data analysis was performed using ChemStation for GC-MS (Agilent, Manchester, UK).

### 2.9. Statistical Analysis

The results are the average of three biological replicates (each replicate consists of leaves from 4–5 cabbage heads) and two technical replicates (*n* = 6). Data obtained were analysed using 2-way ANOVA, with both cabbage accession (or morphotype) and growing condition (glasshouse and field) fitted as treatment effects, and Tukey’s HSD multiple pairwise comparison test used to determine significant differences (*p* < 0.05) between samples. Multifactor analysis (MFA) was used to visualise the GSL and GHP data in a minimum number of dimensions (two or three). All statistical analyses were performed in XLSTAT (version 2019.4.2, Addinsoft, Paris, France).

## 3. Results and Discussion

### 3.1. Effect of Growing Conditions, Cabbage Morphotype and Accession on Myrosinase Activity

**Figure 1:**
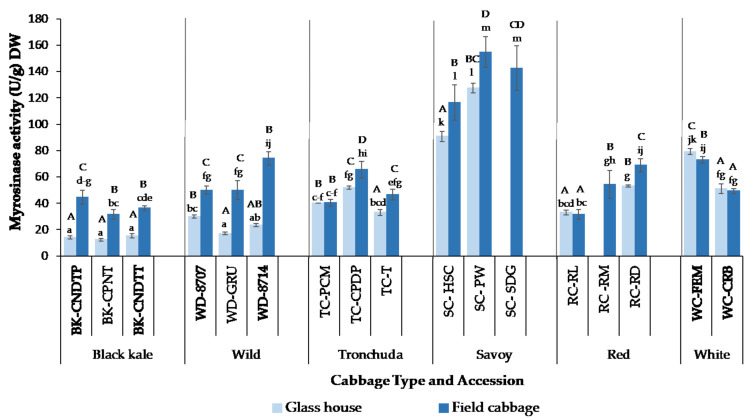
Myrosinase activity of field and glasshouse grown cabbages. Values are means of three biological replicates (each replicate comprising 4–5 cabbage heads) and two separately extracted technical replicates (*n* = 6). Error bars represent standard deviation from mean values. Missing data points implies cabbage accession did not survive under glasshouse growing conditions. Letters “A-D”: bars not sharing a common uppercase letter indicates significant differences (*p* < 0.0001) between accessions and growing conditions within a cabbage morphotype. Letters ”a-k”: bars not sharing a common lowercase letter indicates significant differences (*p* < 0.0001) between accessions and growing conditions between cabbage morphotypes. See Table 1 for full names of cabbage accessions.

The myrosinase activity of cabbages grown on the field and in the glasshouse is shown in Figure 1. Myrosinase activity ranged from 12.2 U/g DW (BK-CPNT) to 127.4 U/g DW (SC-PW) in glasshouse samples and from 31.5 U/g DW (BK-CPNT and RC-RL) to 154.8 U/g DW (SC-PW) in field samples. Growing condition (glasshouse versus field), cabbage morphotype, cabbage accession and the interactions between these parameters significantly (*p* < 0.0001) affected myrosinase activity. The myrosinase activity of cabbage accessions within a cabbage morphotype differed significantly for all cabbage morphotypes studied. This agrees with previous reports that myrosinase activity varies within varieties and plant species [65]. Singh et al. [38] and Penas et al. [29] also reported variations in the myrosinase activity of different cabbage varieties within and between cabbage morphotypes. There were significant differences in the myrosinase activity of field and glasshouse grown cabbages across most of the accessions studied. Field grown cabbages had significantly higher myrosinase activity than glasshouse cabbages, except for WC-FEM, where the myrosinase activity of the glasshouse sample was significantly (*p* < 0.003) higher than that of the field grown counterpart.

The myrosinase activity of TC-PCM, RC-RL and WC-CRB accessions did not differ significantly between field and glasshouse grown cabbages. Authors have previously reported that growing/environmental conditions affect myrosinase activity in *B. oleracea* species [24,25,26,29], and the results obtained from this study agree with their reports. The lower myrosinase activity of glasshouse cabbages might have been due to higher growth temperatures than those grown in the field. Minimum and maximum glasshouse temperatures were 14 and 43 °C, respectively, while minimum and maximum field temperatures were 6 and 24 °C, respectively (Supplementary Table S1). There are several possible reasons for the differences observed. One hypothesis could be that high temperatures reduced myrosinase enzyme synthesis or led to its more rapid denaturation. Another possible reason may have been that the process of synthesis and degradation of the enzyme (turn-over rate) was occurring faster at the higher growth temperatures, meaning that the plant did not accumulate a pool of enzymes at any one time. However, given that we can only see a snapshot in time when plants are sampled for enzyme assays, and each accession was harvested just once at a consistent time of day, it is not possible to infer the kinetics of these reactions occurring within the plant from the data in the present study. The kinetics of myrosinase synthesis and degradation within the plant is an area that warrants further study. Penas et al. [29], in their study of cabbages grown in different parts of Spain, reported that myrosinase activity was lower in cabbages grown in eastern Spain that were exposed to a higher growing temperature when compared to those grown in northern Spain with lower growing temperatures. It is, however impossible to say unequivocally that the lower myrosinase activity observed in the glasshouse samples is as a result of higher growth temperatures and not due to other stress factors, as we were unable to grow the plants in the glasshouse under lower temperatures similar to those observed in the field due to unavailability of cooling facilities within the glasshouse used in the study.

Another possible reason for the significantly lower enzyme activity in glasshouse cabbages could be due to stress factors during growth. Glasshouse cabbages were grown in pots, which may have led to stress from restricted root volume and reduced the amounts of nutrients (sulphur and nitrogen) available, potentially resulting in fewer enzymes and substrates being synthesized. Cabbage grown in the glasshouse achieved a lower above ground biomass than the field grown ones, indicating some form of stress. This was also evident in the differences in size of the closed heart cabbage heads, with the glasshouse plants having smaller heads than the field plants, as reported in Section 2.1. Their leaves appeared to be thinner and less robust than the field cabbages, as is often found in plants grown in protected environments that are not exposed to stimuli, such as wind, which for decades has been known to encourage the formation of thicker cell walls and smaller cells [66]. Hirai et al. [67] found that under nitrogen and/or sulfur limiting growth conditions, genes encoding myrosinase enzyme synthesis were down-regulated in Arabidopsis in order to facilitate storage of these elements in the form of glucosinolates in the leaf tissue. Yuan et al. [68] and Rodríguez-Hernández et al. [69] showed that salt stress reduced myrosinase activity in radish sprouts and broccoli, respectively. Pests and insect attack in field cabbages may have also led to higher myrosinase synthesis and/or accumulation in the cabbages. Accessions that did not show significantly different myrosinase activities between the two growing environments, or in the case of WC-FEM, higher myrosinase activity in glasshouse samples, might have been able to tolerate the glasshouse conditions and may have found it conducive for growth, while accessions that did not survive in the glasshouse may have found the conditions too harsh. Increased myrosinase activity as a result of abiotic stress, such as salt, temperature and drought, has been reported in various *Brassicaceae* species [70,71,72]. Increased myrosinase activity would result in enhanced glucosinolate hydrolysis to beneficial isothiocyanates, which would not only be beneficial to consumers but would also serve as defence compounds for the plants, thereby protecting them against insect and pest attacks.

### 3.2. Protein Content and Specific Myrosinase Activity of Glasshouse and Field Grown Cabbages

The protein content and specific activity of myrosinase for all accessions and growing conditions studied are presented in Table 4. The protein content and specific activity of samples studied were significantly (*p* < 0.05) affected by growing conditions and cabbage accession. Protein content did not correlate with myrosinase activity.

Savoy and white cabbage accessions, which had the highest myrosinase activity, had the lowest protein contents. Just like myrosinase activity, the protein content of glasshouse samples was significantly lower than the field samples. This might be as a result of plant stress during growth, which prevents the plant from producing more nutrients than required or using up its stored nutrients in order to survive, as previously discussed in Section 3.1. Plant proteins have been reported to react negatively to environmental stress [26]. The results obtained are in agreement with Rosa and Heaney [73], who reported higher protein contents in Portuguese cabbage grown in lower environmental temperatures compared to those grown in higher temperatures.

Specific activity of the cabbages was similar to the myrosinase activity and protein content, with field grown cabbages generally having higher specific activity than the glasshouse cabbages. Savoy and white cabbage accessions had significantly higher specific activities than other cabbage morphotypes, as indeed both were found to have significantly higher total myrosinase activity (Figure 1). White cabbage has previously been reported to have higher specific activity than red cabbage [28], which is in agreement with the results of this study. However, a study conducted by Singh et al. [38] showed red cabbage with a higher specific activity than white and savoy cabbage. This might have been due to the differences in varieties studied or protein content of the cabbages, which was not reported in their study.

### 3.3. Effect of Cabbage Morphotype and Accession on GSL Profile and Concentration of Field Grown Cabbages

GSL profiles across cabbage accessions are presented in Figure 1; the statistical output of significant differences within and between cabbage morphotypes is documented in Supplementary Table S2. In total, nine different GSLs were identified across all accessions tested (Table 2): seven aliphatic GSLs, namely sinigrin (SIN), gluconapin (GPN) and epi/progoitrin (PROG), glucoibeverin (GIBVN), glucoerucin (GER), glucoiberin (GIBN) and glucoraphanin (GRPN), and two indole GSLs, glucobrassicin (GBSN) and 4-hydroxyglucobrassicin (4-HOH). PROG, GIBN and GRPN were the most abundant GSLs across all accessions studied, with 4-HOH, GIBVN and GER being the least abundant. 4-HOH was present in negligible amounts (<1.0 µmol/g DW) in all accessions, contributing not more than 1% to the total GSL content of the cabbages. When considering the ratio of total aliphatic to indole GSL concentrations in the accessions, over 60% of total GSL concentration was made up of aliphatic GSLs, with less than 30% from indole GSLs, with the exception of savoy SC-PW accession, where indole GSL comprised 36% of total GSL concentration (Supplementary Table S2).

GSL profiles and concentrations varied across cabbage accessions and differed significantly (*p* < 0.05) in some cases between and within cabbage morphotypes and accessions. Only five of the nine individual GSLs identified in the cabbages studied were found in black kale accessions:—GIBN, GRPN, GBSN, 4-HOH and GER—the last of which was present in BK-CNDTT alone. GRPN was the major GSL present in black kale accessions, consisting of over 50% on average of the total GSL content of black kale. The proportion of GRPN is similar to those previously reported by Kushad et al. [74] but much higher than those reported by Cartea et al. [40]. Previous studies detected SIN and PROG in kale and reported SIN as the main GSL in kale varieties [32,40,74]; however, SIN and PROG were not detected in the current study. There was a significant difference in total and individual GSL concentrations within black kale accessions, except for 4-HOH, which did not differ significantly (*p* = 0.401). BK-CPNT had the highest total GSL content (47.5 µmol/g DW).

GIBVN and GER were not identified in any of the wild and tronchuda cabbage accessions studied, while GIBN and GRPN were identified in all accessions except for WD-8707 accession. The concentration of individual GSLs differed significantly (*p* < 0.0001) across all wild and tronchuda cabbages. PROG and GPN were the most abundant GSLs in WD-8707 and WD-8714, while PROG and GRPN were the most abundant in WD-GRU. In tronchuda cabbages, SIN, GIBN and GBSN were at the highest concentrations, with SIN comprising up to 42% in TC-T.

A previous study [40] on GSL profile and concentrations in tronchuda cabbage identified 14 GSLs, compared to seven found in this study. However, GER was not identified in both studies, and proportions of the individual GSLs identified in both studies were similar.

The total GSL content of wild and tronchuda accessions differed significantly (*p* < 0.01 and *p* < 0.0001, respectively) between accessions within each cabbage morphotype. The most abundant GSLs in savoy cabbages were GIBN, SIN and GBSN, with GIBN concentrations as high as 61.3 µmol/g DW (57% of the total GSLs) in SC-SDG. GER was not identified in savoy accessions, and GPN was present in very low amounts in SC-SDG only. Similar proportions of savoy GSLs were reported by Ciska et al. [41] and Hanschen and Schreiner [43], but in both studies more individual GSLs were identified in the savoy varieties investigated than those reported in this study. For example, both studies identified GER in savoy cabbages, although present in trace amounts in the Ciska et al. [41] study. The total GSL content of savoy cabbages ranged from 47.6 µmol/g DW to 108.5 µmol/g DW. SC-SDG accession had significantly higher (*p* < 0.0001) total GSLs than SC-HSC and SC-PW, with SC-HSC having significantly lower total GSLs than the other two accessions.

In red and white cabbages, PROG, GIBN and GRPN were the most abundant GSLs. GBSN was also abundant in WC-CRB and RC-RL accessions, while GER was not identified in either accession. The concentrations of GRPN, GIBVN and GER did not differ significantly between red cabbage accessions. WC-CRB had significantly higher amounts of SIN, GIBN, GBSN and total GSL compared to WC-FEM, but differences in PROG and GRPN content were not significant. The total GSL content of RC-RL was significantly (*p* < 0.0001) higher than the other two red cabbage accessions. The results obtained agree with those previously reported [22,41,43]. However, a few studies disagree with the findings of this study; a previous study conducted by Park et al. [75] quantifying red cabbage GSL reported SIN absent in red cabbage, while Zabaras et al. [76] found GPN as the most abundant GSL in red cabbage.

**Figure 2:**
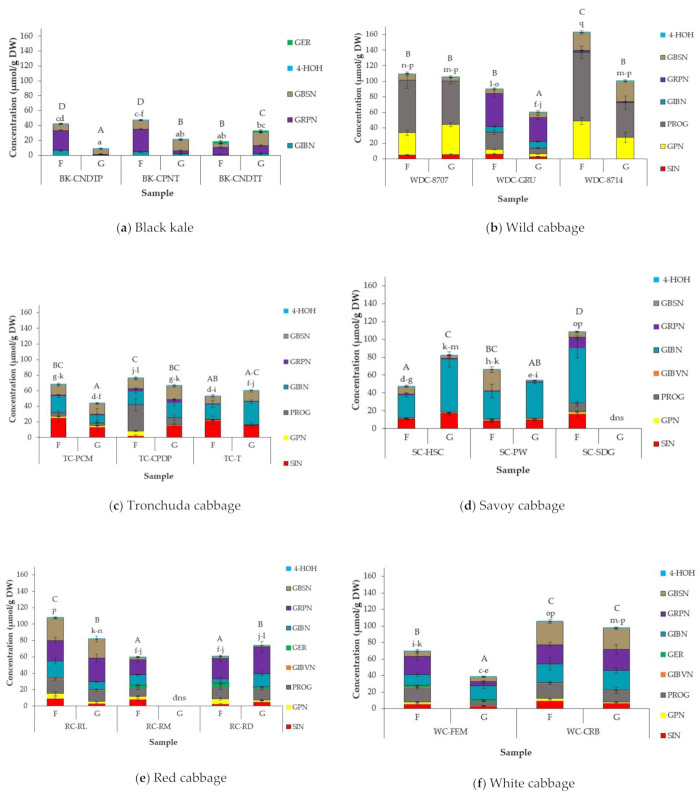
Glucosinolate concentrations (µmol/g DW) in different accessions of (**a**) Black kale; (**b**) Wild cabbage; (**c**) Tronchuda cabbage; (**d**) Savoy cabbage; (**e**) Red cabbage; and (**f**) White cabbage grown in the field and glasshouse. Error bars represent standard deviation from mean values. Letters above bars refer to differences in total GSL concentration. Letters ”A-D”: bars not sharing a common uppercase letter differ significantly (*p* < 0.05) between accession and growing conditions within a cabbage morphotype (i.e., within each separate graph). Letters ”a-q”: bars not sharing a common lowercase letter differ significantly (*p* < 0.0001) between cabbage morphotypes, accessions, and growing conditions (i.e., between the separate cabbage morphotype graphs). Abbreviations: F = Field, G = glasshouse; dns = did not survive. For abbreviations of accessions and compounds see Table 1 (cabbage accessions) and Table 2 (GSLs).

Individual GSLs and total average GSL concentrations differed significantly (*p* < 0.0001) across all accessions, irrespective of cabbage morphotype. Total average GSL concentrations of accessions studied ranged from 18.9 µmol/g DW (BK-CNDTT) to 163.1 µmol/g DW (WD-8714). These differences were due to variations in GSL profiles and concentrations of individual GSLs. Wild cabbages generally had higher total GSL concentrations (Figure 2b) than other cabbage morphotypes, and these high concentrations were driven by significantly higher amounts of PROG in wild cabbages. Lower concentrations of total GSL observed in black kale accessions (18.9 µmol/g DW to 47.5 µmol/g DW) were due to lower numbers and concentrations of individual GSLs compared to the other cabbage morphotypes studied (Figure 2a). The variability in GSL concentrations between and within cabbage morphotypes and accessions is in agreement with previous reports that GSL profiles and concentrations vary between *Brassica* species and varieties [5,29,39,40,43,52,77]. The difference in GSL profiles of *Brassica* vegetables has been linked to genetic factors, while interactions between environmental and genetic factors are largely responsible for differences in GSL concentrations [8]. In general, concentrations of individual and total GSL of the gene bank cabbages reported in this study are much higher than those reported for commercial and gene bank cabbage varieties/accessions in the literature [29,40,41,43,74]. One reason for this may be due to the different varieties/accessions studied, implying that gene banks may indeed be a useful source from which to select accessions with higher GSL concentrations.

Differences in postharvest handling/time could have also contributed to the higher abundance of GSLs observed in the current study. Most varieties used in the literature were obtained from the supermarket and would have gone through a standard commercial supply chain upon harvest, unlike the samples used in this study, which were transferred to the laboratory immediately after harvest. The absence of commercial postharvest storage and handling processes in the current study could account for the differences observed between the samples and those reported in the literature. Total GSL abundance has been shown to decrease in *Brassica* vegetables stored for 7 days at 4–8 °C [78]. Lastly, differences in the conditions under which the plants were grown and/or harvested could also be responsible for the variations in GSL concentrations observed. This suggests that it is not only important that the right accession/variety is selected, but it must also be grown under optimal conditions and given as short a supply chain as possible to achieve optimum GSL abundance in the plants. The higher GSL concentrations in the present study can enhance the potential health benefits that may be derived from their consumption.

The differences in GSL profiles and concentrations of the accessions studied can potentially influence the sensory and health properties of the cabbages. For example, the absence of SIN and PROG in black kale accessions and higher concentrations of PROG reported in wild cabbage accessions may potentially influence the sensory characteristics of these cabbages, given SIN and PROG have been linked with bitter taste in *Brassica* vegetables [22,79]. On the other hand, higher amounts of GRPN (the precursor GSL for SFP formation linked to several health promoting properties of *Brassicas*) in kale, red and white cabbages could enhance the potential health benefits derived from their consumption [80]. The differences in cabbage accessions, growing conditions and geographical location, as well as environmental factors during cabbage cultivation, all play a vital role in GSL profile and concentration and therefore make comparing results between different studies difficult.

### 3.4. Effect of Growing Conditions on GSL Concentrations in Cabbage Accessions

The effect of growing conditions on GSL concentration is presented in Figure 1, with significant differences within and between cabbage morphotypes presented in Supplementary Table S2. While the GSL profile of cabbage accessions studied did not differ between growing conditions, there was a difference in GSL abundance between glasshouse and field grown cabbages. Total GSL concentrations in field grown samples ranged from 18.9 µmol/g DW (BK-CNDTT) to 163.1 µmol/g DW (WD-8714) and glasshouse samples from 8.81 µmol/g DW (BK-CNDTP) to 105.5 µmol/g DW (WD-8707). WD-8714 had significantly (*p* < 0.0001) higher concentrations of total GSLs compared to all other accessions, and this was largely due to the abundance of PROG and GPN, making up 83% and 69% of total GSLs in field and glasshouse samples, respectively.

Cabbages grown in the field had higher total GSL concentrations than glasshouse samples across most accessions studied, with a few exceptions (BK-CNDTT, TC-T, SC-HSC, and RC-RD), where total GSL concentrations were higher in glasshouse samples. These differences were significant in some but not all cases. Growing conditions significantly affected individual GSL concentrations between and within cabbage morphotypes and accessions. With the exception of black kale accessions, both field and glasshouse cabbages were predominantly abundant in aliphatic GSLs, with averages of 82 and 78%, respectively across all accessions, while indole GSLs comprised only 18 and 22% of total GSLs in field and glasshouse samples, respectively. In black kale accessions, however, growing conditions seemed to influence the ratio of aliphatic to indole GSL present in the samples. All black kale accessions grown in the glasshouse had much higher total indole GSL, with up to seven-fold differences reported in BK-CNDTP samples (Supplementary Table S2). The differences observed are mainly due to differences in the ratio of individual aliphatic to indole GSL present in the samples and not higher concentrations of indole GSLs in the glasshouse samples, as there was no significant difference observed in the concentrations of the most abundant indole GSL, GBSN, present in the samples between growing conditions (except for BK-CNDTT).

There was no clear pattern for the abundance of individual GSLs, as some GSLs were significantly higher in glasshouse samples for some accessions, but lower or not significantly different in others. PROG and GRPN were either significantly higher in field samples or did not significantly differ from glasshouse samples within accessions, except for RC-RD accession, where GRPN was significantly higher (*p* < 1.0001) when grown in the glasshouse. GRPN abundance in BK-CNDTP and BK-CPNT field grown accessions was up to 90% more than the corresponding glasshouse grown cabbages. GBSN was the most stable GSL across growing conditions, as there was no significant difference (*p* = 0.101) in GBSN between field and glasshouse cabbages.

Growing conditions such as growth temperature and photoperiod have been shown to influence the abundance of GSLs. There are several possible reasons for the differences observed in GSL concentrations in the different growing conditions. The higher total GSL content reported in most field samples could be due to production of higher amounts of GSLs by the plant in response to insect and pest attack on the field when compared to glasshouse samples. GSL compounds are plant metabolites produced by plants for defence against stress and attack from insect and pests [8,81]. In addition, the higher amount of GSLs in field samples could also be due to the lower average temperatures during growth (6 to 24 °C) compared to the higher temperatures in the glasshouse (14 and 43 °C) (Supplementary Table S1). Growth temperatures have been reported to influence GSL concentrations in *Brassica* vegetables. *Brassica* vegetables are generally thought to be cool weather crops, with average growing temperatures between 4–30 °C [82]. The optimum temperature for growth varies between different types of *Brassicas* and going below or above that temperature could affect GSL concentrations. The exact mechanism of GSL biosynthesis under different temperature conditions is unclear because of several interacting factors, such as drought and photoperiod, but it has been reported that plant stress due to high or low growing temperatures may enhance activities of transcription factors such as *MYC2* and *MYB28*, which promote GSL biosynthesis [42,83]. Literature studies have, however, generally reported higher GSLs at higher growing temperatures; Rosa and Rodrigues [27] reported a higher GSL content in young cabbage plants when grown at 30 °C compared to 20 °C. Lower GSL concentrations was reported in kale grown at lower temperatures compared to those grown at higher temperatures [32,33]. In addition, several authors have reported higher GSL concentrations in spring/summer grown cabbages (average temperatures between 25–30 °C) compared to autumn grown plants (temperatures < 20 °C) [29,39,40,41]. The lower amounts of GSL accumulated in glasshouse plants could also be the result of plant growing conditions. Glasshouse samples were grown in pots with drainage holes to allow excess water to seep out. However, this could have also led to sulphur leaching, leading to sulphur deficiency in the soil, and plants were not fed with sulphur fertilizers. Sulphur is a major precursor for GSL biosynthesis, and its deficiency has been reported to reduce GSL concentrations in *Brassica* plants, especially aliphatic GSLs, as sulphur deficiency limits methionine synthesis (basic substrate for aliphatic GSL biosynthesis) as opposed to tryptophan, a non-sulphur amino acid and precursor for indole GSL biosynthesis [84]. On average, reduced amounts of aliphatic GSLs were accumulated in glasshouse plants compared to field plants, while glasshouse samples accumulated higher amounts of indole GSLs than field samples. Sulphur was reported to influence the aliphatic GSL concentrations in rapeseed more than indole GSL [84]. However, glasshouse plants, which had significantly higher GSL concentrations compared to their field counterparts, may have found the glasshouse conditions more favourable than other accessions, which resulted in enhanced GSL production.

The results of this study show that cabbages differ in their requirements for growth, and it is important to plant cabbage accessions in growing conditions that are best suited for their maximum development, as individual plants respond differently under different environmental conditions. Optimizing agronomy practices and applying limited abiotic stress in a controlled manner could be a way of increasing myrosinase activity and GSL production in some *Brassica* species.

### 3.5. Effect of of Cabbage Morphotype and Accession on Glucosinolate Hydrolysis Products (GHPs) of Field Grown Cabbages

A total of 22 GHPs were identified and quantified from the cabbage accessions studied, comprising 11 ITCs and 11 nitriles/epithionitriles (Table 3). Concentrations of GHPs are presented in Figure 2, with significant differences between and within cabbage morphotypes and accessions presented in Supplementary Table S3. Results are expressed as sulforaphane equivalents.

**Figure 3:**
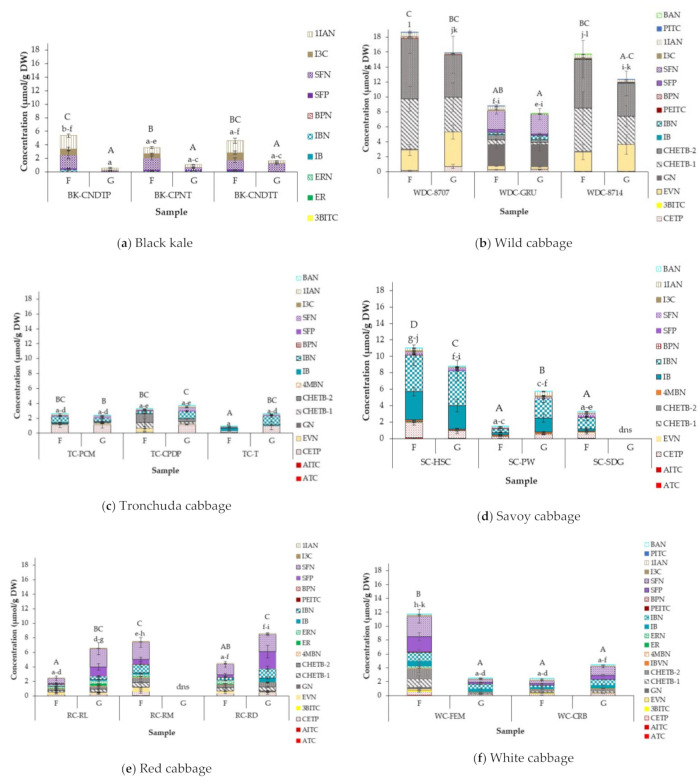
Glucosinolate hydrolysis products (GHPs) (µmol/g DW) in different accessions of (**a**) Black kale; (**b**) Wild cabbage; (**c**) Tronchuda cabbage; (**d**) Savoy cabbage; (**e**) Red cabbage; and (**f**) White cabbage grown in the field and glasshouse. Error bars represent standard deviation from mean values. Letters above bars refer to differences in total GHP concentration. Letters “A-D”: bars not sharing a common uppercase letter differ significantly (*p* < 0.05) between accessions and growing conditions within a cabbage morphotype (i.e., within each separate graph). Letters ”a-l”: bars not sharing a common lowercase letter differ significantly (*p* < 0.0001) between cabbage morphotypes, accessions, and growing conditions (i.e., between the separate graphs). Compounds with colour shades similar to one another are GHPs of corresponding GSLs presented in Figure 2. Abbreviations: F = Field; G = glasshouse; dns = did not survive. For abbreviations of accessions and compounds see Table 1 (cabbage accessions) and Table 3 (GHPs).

The type and concentration of GHPs formed differed between cabbage accessions. Predominant GHPs did not differentiate between accessions within a cabbage morphotype but varied across cabbage morphotypes. There was a significant difference in the concentrations of individual and total GHPs formed within and between cabbage morphotypes and accessions (Figure 3 and Supplementary Table S3). Wild cabbage accessions had the highest levels of GHPs formed (8.79 µmol/g DW—8.6 µmol/g DW; Figure 2b) and tronchuda accessions the lowest (0.95 µmol/g DW—3.27 µmol/g DW; Figure 2c).

GHPs of GRPN and GBRN were the main GHPs detected in black kale accessions, with nitrile concentrations accounting for 74–89% of the total GHPs. BK-CPNT accessions had significantly lower total GHPs than BK-CNDTP. Isomers of CHETB, nitriles of PROG hydrolysis, were the most abundant GHPs formed in wild cabbages, except for WD-GRU, which had higher amounts of GN (PROG ITC) compared to the nitriles formed. This was unexpected, and it is unclear why this happened, because more nitriles than ITCs were formed for other GSLs present in the same sample. A possible explanation for this could be the activity of epithiospecifier modifier proteins (ESMs), enhancing the activity of specific myrosinase isoenzymes, which hydrolyse PROG present in the samples. ESM inhibits the activity of ESP, preventing the formation of nitriles and epithionitriles, and instead promotes ITC formation [15,85,86]. GN have been associated with bitter taste [87] and adverse effects on thyroid metabolism, leading to goitre formation. The reports on goitre formation are limited and based on animal studies, which show that average daily intake is not enough to produce adverse effects in humans [8]. However, to limit the health risks, genetic manipulation and selective breeding methods used to increase GRPN contents by threefold in ‘*Beneforte*’ broccoli [88] could be employed to reduce PROG contents in the wild accessions. The main GHPs of tronchuda accessions were CETP and IBN, nitriles of SIN and GIBN, respectively. Total GHPs of TC-CPDP were significantly higher than TC-T. IBN and IB (GIBN hydrolysis products) were the most abundant GHPs in savoy cabbages, and SFP and SFN (hydrolysis products of GRPN) the most abundant in red and white cabbages.

In savoy, SC-HSC varied significantly from SC-PW and SC-SDG accessions, containing up to 60% more GHPs than the other two accessions. The much lower concentrations of GHPs in SC-PW compared to SC-HSC were unexpected due to similar concentrations of GSLs in both accessions. A similar trend was noticed between WC-CRB and WC-FEM accessions, where much lower GHPs were formed in WC-CRB accession, with significantly higher GSLs than WC-FEM. This might be related to variation in myrosinase and ESP activities within the samples. As previously discussed in Section 3.1, WC-FEM had significantly higher myrosinase activity than WC-CRB (Figure 1), which may explain the higher concentrations of GHPs formed. However, this is not the case in savoy cabbages, as SC-PW had the highest myrosinase activity (see Figure 1). It is hypothesized that myrosinase isoenzymes and ESP of SC-PW accession may be less stable than the other accessions and was, therefore, denatured before permitting full hydrolysis. As previously discussed, ESM activities promoting ITC formation may also be responsible for the higher GHP concentrations observed. For example, although GIBN concentration in WC-FEM was significantly (*p* < 0.0001) lower than that of WC-CRB, the amount of IB, the ITC formed from GIBN, was significantly (*p* < 0.0001) higher in WC-FEM than in WC-CRB. Another possible reason for the variation in GHP concentrations could be due to the type of myrosinase isoenzyme present within the samples. It has been reported that myrosinase isoenzymes differ in the rate at which they hydrolyse individual GSLs, though little is known of their substrate specificity. James and Rossiter [89] found that in the presence of ascorbic acid, two myrosinase isoenzymes identified in *Brassica napus* L. differed in the way they degraded SIN and neoglucobrassicin (NEO), with SIN being degraded more rapidly than NEO by both isoenzymes under the same conditions. While there are limited studies on the conversion ratio of GSLs to GHPs, studies on GHP formation in *Brassica oleracea* [43] and rocket salad [42] have shown that conversion of GSLs to GHPs is not always a linear reaction and GHP concentrations are generally much lower than the precursor GSL concentrations.

Several GHPs were identified in cabbage accessions where their GSLs were not detected: tiny amounts of 3BITC (GPN hydrolysis product) were formed in BK-CNDTT; 4MBN (nitrile of GIBVN) in tronchuda; EVN (GPN nitrile) in savoy cabbages; and ER and ERN (GER GHPs) in red and white cabbages. PEITC and BPN (GHPs of gluconasturtiin), PITC and BAN were also formed in most accessions. This could be due to concentration of the respective GSLs being below the limits of detection of the LC-MS^2^ instrument used. A previous study of turnips detected GHPs of glucoberteroin, though the intact GSL was not detected [90]. A recent study on horseradish, wasabi, watercress, and rocket also detected GHPs, where their intact glucosinolates were not identified [91]. The profile of GHPs in this study is in agreement with the study of Hanschen and Schreiner [43]. However, in their study, they found CETP (nitrile from SIN hydrolysis) as the main GHP in savoy, red and white cabbages, which is inconsistent with this study, where GIBN GHPs (IB and IBN) and GRPN GHPs (SFP and SFN) were the main compounds detected. This difference can be attributed to the different varieties/accessions studied.

In general, the relationship between individual GSLs and their corresponding GHPs within an accession was as expected, where the dominant GSL resulted in their corresponding dominant GHPs, which is helpful in confirming the efficiency and accuracy of the GHP extraction method. Overall, nitriles and epithionitriles were the major hydrolysis products formed across all cabbage accessions, as has been reported previously in raw cabbage [62,92]. This is due to the activity of ESP and other nitrile forming proteins present in the samples, which hydrolyse GSLs to epithionitriles and nitriles instead of the more beneficial ITCs [92].

### 3.6. Effect of Growing Condition on GHP Concentrations

GHP profile and concentration in the two different growing conditions studied is presented in Figure 3, with the significant differences between growing conditions reported in Supplementary Table S3. The profile of the GHPs detected were similar between growing conditions, with a few exceptions. For example, BPN was identified in black kale field samples but not detected in glasshouse samples. GHP concentrations in field and glasshouse ranged from 0.95 µmol/g DW (TC-T) to 18.6 µmol/g DW (WD-8707) and 0.59 µmol/g DW (BK-CNDTP) to 15.9 µmol/g DW (WD-8707), respectively. Within accessions, total GHP accumulation was significantly higher in field plants than glasshouse, except for wild cabbage accessions, TC-PCM and WC-CRB, where total GHPs were higher in glasshouse samples; however, the differences were not significant, except in WC-CRB, where a significant difference was observed. Generally, total GHP concentrations followed a similar pattern to total GSLs, with a few exceptions. For example, the BK-CNDTT glasshouse sample had significantly lower total GHPs compared to the field sample (Figure 3a), despite the significantly higher total GSL in the glasshouse sample (Figure 2a). Significantly higher myrosinase activity, and possibly ESP activity, in the BK-CNDTT field compared to glasshouse sample may have led to the formation of more GHPs (Figure 1). A similar trend was observed in savoy accessions, where an abundance of GSL under one growing condition did not necessarily result in higher amounts of GHP formed. The results obtained in our study are in agreement with those reported by Jasper et al. [42], where growth temperatures had different effects on the amount of GSL and GHPs formed in rocket salads.

In summary, the results of this study show the importance of having both high myrosinase activity and GSL accumulation in plants, as they have a direct impact on the amount of hydrolysis compounds formed. It is therefore important to ensure that cabbages are cultivated under optimised growing conditions (such as temperature, available sulphur/nitrogen and controlled biotic stress) that favour both high myrosinase and GSL accumulation and not only one or the other.

### 3.7. Multifactor Analysis (MFA) of GSLs and GHPs Identified in Cabbage Accessions Grown under Two Different Conditions

**Figure 4:**
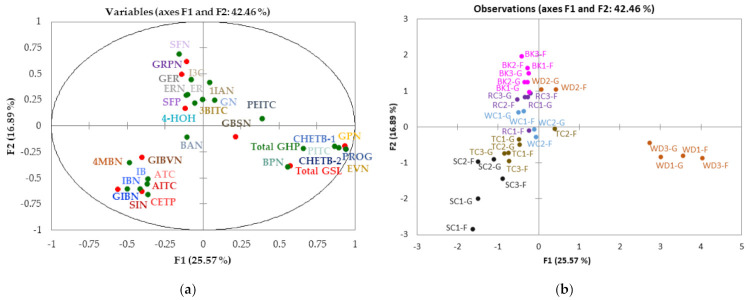
MFA map of glucosinolates and glucosinolate hydrolysis products (**a**) distribution of variables and (**b**) sample distribution. For codes and distribution on plot, refer to Table 1 (cabbage accessions) and Table 2 and Table 3 (compounds). Compounds with different shades of the same colour in Figure 3a refer to the GSL and corresponding GHP. Key: F = Field ; G = Glasshouse; ● GSL = Glucosinolates; ● GHPs = Glucosinolate hydrolysis products; ● BK= Black kale; ● WD = Wild cabbage; ● TC= Tronchuda cabbage; ● SC = Savoy cabbage; ● RC = Red cabbage; ● WC = White cabbage.

To investigate the underlying structure of the results, MFA was performed on the GSL and GHP data from the cabbage accessions. Figure 4 shows distribution of the cabbage accessions as well as the scores and loadings of MFA performed on the mean data of GSLs and GHPs. Dimensions 1 and 2 (F1, F2) explained 42% of the variance in the data, but other dimensions did not provide any new information; therefore, only F1 and F2 are presented and discussed. The plot demonstrates that individual GSLs were positively correlated with their corresponding GHPs. From the plot, it is clear that cabbages were mostly distinguished based on morphotype rather than accessions or growing conditions, except for wild cabbage accessions, where there was a clear separation of WD2 (WD-GRU) from WD1 (WD-8707) and WD3 (WD-8714).

Based on the MFA, samples were grouped into three distinct clusters: one cluster comprised of black kale, red cabbage, white cabbage and WD-GRU accessions, another tronchuda and savoy cabbage accessions, and the final cluster WD-8707 and WD-8714 accessions. Black kale, red cabbage, white cabbage and WD-GRU correlated positively with GRPN, GER, 4-HOH and their hydrolysis products. Tronchuda and savoy cabbage samples correlated positively with GIBN, GIBVN, SIN and their hydrolysis products. WD1 and WD2 correlated positively with GPN and PROG and their nitriles, as well as total GSLs and GHPs, but was negatively correlated with black kale, red cabbage, white cabbage and WD-GRU accessions. An additional Pearson correlation demonstrating significant correlations (*p* < 0.05) between various GSLs and GHPs is presented in Supplementary Table S4. GIBN correlated negatively (r^2^ > −0.3; *p* < 0.01) with PROG and its hydrolysis products, GPN and its hydrolysis products, and PITC. On the contrary, GPN was strongly positively correlated (r^2^ > 0.6; *p* < 0.0001) with PROG and its hydrolysis products, EVN, PITC, total GSL and total GHPS. Total GSLs were significantly positively correlated (r^2^ = 0.5; *p* < 0.01) with total GHPs. Strong significant positive correlations (r^2^ > 0.5; *p* < 0.05) were observed between individual GSLs and their corresponding GHPs. For example, GRPN was positively correlated with SFP and SFN (r^2^ > 0.5 and 0.8; *p* < 0.01 and *p* < 0.0001 respectively).

It is obvious that the separations observed between samples are mainly driven by differences in GSLs and GHPs most accumulated in the samples: GN, GRPN, GER, 4-HOH and their GHPs in black kale, red cabbage, white cabbage and WD-GRU accessions; GIBN, SIN, GIBVN and their GHPs in tronchuda and savoy cabbage accessions; and lastly, PROG, GPN and their GHPs in WD-8707 and WD-8714 accessions. WD-8707 and WD-8714 had the highest concentration of total GSLs and GHPs, and this was responsible for the positive correlation of these accessions to total GSLs and GHPs observed. It is worth mentioning that PROG and CHETB, which were largely responsible for the high concentrations of total GSLs and GHPs in these accessions, correlated positively with total GSLs and GHPs. The result obtained provides a clear picture of the similarities and differences in GSL and GHP profile and concentrations of the different cabbage morphotypes and accessions studied.

Like any other study, some limitations were encountered in this study. First, the cabbage seeds used in the study were obtained from a gene bank. This means they have not been bred for uniformity in terms of plant characteristics and abundance of phytochemical compounds. Breeding programmes to date have mostly focused on developing disease-resistant and environmentally resilient crops, with less emphasis on the content of phytochemical compounds. This implies that there may be large variations in phytochemical compounds between cabbage heads/plants of the same accession, as has been observed in *Marathon* broccoli heads [93], and this may have influenced the results obtained in the present study. To reduce the effects of possible variation between plant heads, four to five heads were mixed together to obtain a representative sample. However, considering the amounts of heads used during the study, some variations may still have existed within the samples.

Second, the GC-MS method used for GHP analysis was long and required several steps to ensure that all GHPs present in the sample could be identified. However, some GHPs may have been lost or converted into other compounds in the process due to their very volatile and unstable nature. Though care was taken during the analysis to prevent losses, the rigorous analytical method may have led to some losses of the more volatile compounds.

## 4. Conclusions

In line with the primary hypothesis of the study, the results demonstrated that myrosinase activity as well as profiles and concentrations of GSLs and GHPs were all influenced by growing conditions, cabbage morphotypes and accession. However, in agreement with our secondary hypothesis, the profile and concentration of GSLs and GHPs formed were substantially more influenced by cabbage morphotype than accession. The study showed that planting cabbages in high growth temperatures and stressful conditions resulted in lower myrosinase activity. Myrosinase activity differed between accessions and cabbage morphotypes, although morphotype tended to have the more significant impact. Savoy cabbage accessions had the highest myrosinase activity, while black kale accessions had the lowest myrosinase activity.

The concentration and profile of GSL and GHP compounds accumulated differed between growing conditions and accessions, within and across cabbage morphotypes. While genetic factors had more influence on the GSL profile of the cabbages, differences in the GSL concentration were more affected by environmental factors during growth, which agrees with previous studies [8]. Growing conditions and cabbage accessions seem to have different effects on GSL and GHP formation, with higher GSL concentrations observed within a growing condition or accession not always resulting in a corresponding greater accumulation of GHPs and vice versa. Results obtained from the study showed that a possible reason for the higher GHP concentrations could be higher myrosinase activities in accessions with lower GSLs, as was observed in white cabbage and black kale accessions. However, this this was not the case in all accessions, suggesting there may be other reasons for the differences obtained. The results obtained therefore suggest that it would be incorrect to assume that higher myrosinase activity and/or GSL accumulation would automatically always result in high concentrations of GHPs.

Variations in the GSL and GHP contents imply differences in the potential health-promoting and sensory characteristics of the cabbages studied. For example, the high amounts of SFP present in red and white cabbages could potentially provide more health benefits on consumption when compared to other accessions. Conversely, high concentrations of PROG and GN (compounds linked to bitter taste) in wild accessions may reduce consumer acceptance and liking. However, the contents of GSLs and ITCs in *B. oleracea* vegetables alone does not provide a clear picture of the sensory characteristics of *B. oleracea* vegetables, as other compounds in the plant matrix, such as sugars and sweet tasting amino acids, can influence and modulate the sensory perception of these vegetables, as has been shown in previous studies on *Brassicas* and other crops such as lettuce [22,94,95,96].

Field grown cabbages had much higher GSLs and GHPs than glasshouse plants, with a few exceptions (SC-HSC and RC-RD). However, the biggest differences observed were between cabbage morphotypes, irrespective of the conditions under which they were grown. The result of this study suggests that cabbage morphotype and accession might be more important factors for GSL and GHP profiles of plants than the conditions under which they are grown. All individual GSLs and their corresponding GHPs were identified in the accessions studied, and a correlation between GSLs and GHPs was found. The difference in myrosinase activity and GSL and GHP concentrations could not be linked to morphology of head formation (closed heart or open leaf). The influence of growing conditions on cabbage biochemistry will be an important consideration, as the use of highly protected environments for crop production becomes more prevalent through indoor farming, which will also lead to breeding of cabbages with more compact morphology. Our data indicate that protected conditions need to be optimised, possibly by inclusion of controlled abiotic stress, in order to generate the GSL abundance that is observed in field grown crops.

Aliphatic GSLs, nitriles and epithionitriles were the most abundant compounds identified. The results suggest that consumption of raw cabbage may provide limited health benefits, as more nitriles and epithionitriles are formed than the more beneficial ITCs. It is therefore recommended to process the cabbages in ways that ensure hydrolysis of GSL to ITCs rather than nitriles. Despite the high amounts of nitriles and epithionitriles formed overall, high amounts of health beneficial SFP were detected in some red and white cabbage accessions. The result suggests that some gene bank accessions can be a good source of beneficial compounds and could be used in breeding programmes to introgress areas of the genome that regulate these compounds from the gene bank accessions into elite commercial cultivars. This can also be helpful for selection of more beneficial accessions for commercial cultivation and production. Given that accessions with lower GSL concentrations and higher myrosinase resulted in high GHP concentrations for some of the accessions studied, breeding programmes should not on only focus on selection of accessions with high GSL concentration but should also consider accessions that have high myrosinase activity and ESM, if maximum conversion of GSLs to ITCs is to be achieved.

## Figures and Tables

**Table 1:** Origin and botanical and common names of cabbage accessions planted between May and November 2015.

Genus/Morphotype ^a^	Accession Name	Accession Code	Common Name	Origin	Head Formation
**Black kale**					
*Brassica oleracea* var. *acephala*	Cavolo nero di toscana o senza palla	BK-CNDTP (BK1)	Fodder black kale	Italy	Open leaf
*Brassica oleracea* var. *acephala*	Cavolo palmizio	BK-CPNT (BK2)	Black kale	Italy	Open leaf
*Brassica oleracea* var. *acephala*	Cavolo nero di toscana o senza testa	BK-CNDTT (BK3)	Fodder black kale	Italy	Open leaf
**Wild**					
*Brassica oleracea* var. *oleracea*	Wild cabbage	WD-8707 (WD1)	Wild cabbage	Great Britain	Open leaf
*Brassica oleracea* var. *oleracea*	Wild cabbage	WD-GRU (WD2)	Wild cabbage	New Zealand	Open leaf
*Brassica oleracea* var. *oleracea*	Wild cabbage	WD-8714 (WD3)	Wild cabbage	Great Britain	Open leaf
**Tronchuda**					
*Brassica oleracea* var. *tronchuda*	Penca mistura	TC-PCM (TC1)	Tronchuda cabbage	Portugal	Open leaf
*Brassica oleracea* var. *tronchuda*	Penca povoa	TC-CPDP (TC2)	Tronchuda cabbage	Portugal	Open leaf
*Brassica oleracea* var. *tronchuda*	Tronchuda	TC-T (TC3)	Tronchuda cabbage	Portugal	Open leaf
**Savoy**					
*Brassica oleracea* var. *capitata*	Hybrid savoy wirosa cabbage	SC-HSC (SC1)	Hybrid savoy cabbage	Great Britain	Closed heart
*Brassica oleracea* var. *capitata*	Pointed winter	SC-PW (SC2)	Savoy cabbage	Great Britain	Closed heart
*Brassica oleracea* var. *capitata*	Dark green	SC-SDG (SC3)	Savoy cabbage	Italy	Closed heart
**Red**					
*Brassica oleracea* var. *capitata*	Red langendijker	RC-RL (RC1)	Red cabbage	Great Britain	Closed heart
*Brassica oleracea* var. *capitata*	Rocco marner (Hybrid)	RC-RM (RC2)	Hybrid red cabbage	Great Britain	Closed heart
*Brassica oleracea* var. *capitata*	Red Danish	RC-RD (RC3)	Red cabbage	Netherlands	Closed heart
**White**					
*Brassica oleracea* var. *capitata*	Early market	WC-FEM (WC1)	White spring cabbage	Great Britain	Closed heart
*Brassica oleracea* var. *capitata*	Couve repolho	WC-CRB (WC2)	White cabbage	Portugal	Closed heart
*Brassica oleracea* var. *capitata*	De louviers	WC-DLI (WC3)	Hybrid white cabbage	Great Britain	Closed heart

^a^ Names in bold refer to cabbage morphotype

**Table 2:** Intact glucosinolates identified in cabbage accessions analysed by LC-MS.

Common Name	Chemical Name	Abbreviation	Mass Parent Ion	MS^2^ Spectrum Ion (Base Ion in Bold) ^a^	Reference
sinigrin	2-propenyl(allyl) GSL	SIN	358	278, 275, **259**, 227, 195, 180, 162	[54,55]
gluconapin	3-butenyl GSL	GPN	372	292, 275, **259**, 195, 194, 176	[54,56]
epi/progoitrin	(R, S)-2-hydroxy-3-butenyl GSL	PROG	388	332, 308, 301, 275, **259**, 210, 195, 146, 136	[54,55,56]
glucoiberverin	3-(methylthio)propyl GSL	GIBVN	406	326, 275, **259**, 288, 228,195	[52,54,55]
glucoerucin	4-(methylthio)butyl GSL	GER	420	340, 291, 275, **259**, 227, 195, 178, 163	[52,54,55]
glucoiberin	3-(methylsulfinyl) propyl GSL	GIBN	422	407, **358**, 259	[54,55,56]
glucoraphanin	4-(methylsulfinyl) butyl GSL	GRPN	436	422, **372**, 291, 259, 194	[52,54,55]
glucobrassicin	3-indolylmethyl GSL	GBSN	447	275, **259**, 251, 205	[54,55,56]
4-hydroxyglucobrassicin	4-hydroxy-3-indolylmethyl GSL	4-HOH	463	383, **285**, 267, 259, 240, 195	[54,55,56]

Key: GSL = glucosinolate; ^a^ Base ion highlighted in bold

**Table 3:** Glucosinolate hydrolysis products identified in cabbage accessions analysed by GC-MS.

Precursor Glucosinolate	Glucosinolate Hydrolysis Product		Abbreviation	LRI ^a^	ID ^b^	MS^2^ Spectrum Ion (Base Ion in Bold) ^c^	Reference
	Common name	Chemical Name					
sinigrin	allyl thiocyanate	2-propenyl thiocyanate	ATC	871	B	99, 72, 45, 44, **41**, 39	[58]
	allyl-ITC	2-propenyl isothiocyanate	AITC	884	B	**99**, 72, 71, 45, 41, 39	[58,59]
	1-cyano-2,3-epithiopropane	3,4-epithiobutane nitrile	CETP	1004	B	**99**, 72, 66, 59, 45, 41, 39	[58]
gluconapin	3-butenyl-ITC	1-butene, 4-isothiocyanate	3BITC	983	B	113, 85, **72**, 64, 55, 46, 45, 41	[58,59,60]
	4,5-epithiovaleronitrile	1-cyano-3,4-epithiobutane	EVN	1121	B	**113**, 86, 80, 73, 60, 45	[60]
progoitrin	goitrin	5-vinyloxazolidin-2-thione	GN	1545	B	**129**, 86, 85, 68, 57, 45, 43, 41, 39	[61]
	1-cyano-2-hydroxy-3,4-epit-hiobutane isomer 1	2-hydroxy-3,4-epithiobutylcyanide diastereomer-1	CHETB-1	1225	B	129, 111, 89, 84, 68, **61**, 58, 55, 45	
	1-cyano-2-hydroxy-3,4-epit-hiobutane isomer 2	2-hydroxy-3,4-epithiobutylcyanide diastereomer-2	CHETB-2	1245	B	129, 111, 89, 84, 68, **61**, 58, 55, 45	
glucoiberverin	4-methylthiobutyl nitrile	4-methylthio butanenitrile	4MBN	1085	B	115, 74, 68, **61**, 54, 47, 41	[58]
glucoerucin	erucin	4-(methylthio)-butyl-ITC	ER	1427	B	161, 146, **115**, 85, 72, 61, 55	[58,59]
	erucin nitrile	1-cyano-4-(methylthio) butane	ERN	1200	B	129, 87, 82, **61**, 55, 48, 41, 47	[58,59] [58]
glucoiberin	iberin	3-methylsulfinylpropyl-ITC	IB	1617	B	163, 130, 116, 102, 100, 86, **72**, 63, 61,41	
	iberin nitrile	4-methylsulfinylbutanenitrile	IBN	1384	B	**131**, 78, 64, 47, 41	[58] [58]
gluconasturtin	2-phenylethyl-ITC	2-isothiocyanatoethyl benzene	PEITC	1458	B	163, 105, **91**, 65, 51, 40	
	benzenepropanenitrile	2-phenylethyl cyanide	BPN	1238	B	131, **91**, 85, 65, 63, 57, 44, 51	[60]
glucoraphanin	sulforaphane	4-methylsulfinylbutyl-ITC	SFP	1757	A	160, 114, 85, **72**, 64, 63, 61, 55. 41, 39	[57,59]
	sulforaphane nitrile	5-(methylsulfinyl) pentanenitrile	SFN	1526	B	145, 128, 82, 64, **55**, 41	[57,59] [61]
glucobrassiccin	indole-3-carbinol	1H-indole-3-methanol	I3C	1801	B	**144**, 145, 116, 108, 89	
	indoleacetonitrile	1H-indole-3-acetonitrile	1IAN	1796	B	**155**, 145, 144, 130, 116, 89, 101, 63	[62]
pentyl glucosinolate	pentyl-ITC	1-isothiocyanato-pentane	PITC	1165	B	129, 114, 101, 96, 72, 55, **43**, 41, 39	[63]
glucotropaeolin	benzeneacetonitrile	2-phenylacetonitrile	BAN	1137	A	**117**, 90, 89, 77, 63, 51	[64]

Key: ITC—isothiocyanate. ^a^ Linear retention index on a HP-5MS non-polar column. ^b^ A, mass spectrum and LRI agree with those of authentic compound; B, mass spectrum agrees with reference spectrum in the NIST/EPA/NIH mass spectra database and that in the literature. ^c^ Base ion highlighted in bold.

**Table 4:** Protein content ((mg/g ± SD) DW) and specific activity ((U/mg soluble protein ± SD) DW) of cabbage accessions grown in the glasshouse and on the field.

Cabbage Morphotype/Accession	Protein Content (mg/g ± SD) DW		Specific activity (U/mg Soluble Protein ± SD) DW	
	Glasshouse	Field	Glasshouse	Field
**Black Kale**				
BK-CNDTP	29.1 ± 0.4 ^gh, B^	33.7 ± 0.6 ^l, C^	0.5 ± 0.0 ^a, A^	1.3 ± 0.2 ^d-h, C^
BK-CPNT	24.5 ± 0.1 ^e, A^	35.4 ± 1.0 ^m, D^	0.5 ± 0.1 ^a, A^	0.9 ± 0.1 ^a-d, B^
BK-CNDTT	25.4 ± 3.9 ^e, A^	36.7 ± 0.7 ^m, E^	0.6 ± 0.1 ^ab, A^	1.0 ± 0.0 ^b-e, B^
**Wild**				
WD-8707	27.4 ± 0.7 ^f, C^	31.4 ± 0.1.2 ^jk, E^	1.1 ± 0.1 ^c-f, B^	1.6 ± 0.1 ^ghi, C^
WD-GRU	25.3 ± 0.1 ^e, B^	29.9 ± 0.6 ^hi, D^	0.7 ± 0.1 ^abc, A^	1.7 ± 0.2 ^hij, C^
WD-8714	18.4 ± 0.1 ^a, A^	30.6 ± 0.8 ^ij, DE^	1.3 ± 0.1 ^d-h, B^	2.4 ± 0.2 ^l, D^
**Tronchuda**				
TC-PCM	32.8 ± 0.1 ^kl, D^	33.6 ± 0.2 ^l, E^	1.2 ± 0.0 ^d-h, AB^	1.2 ± 0.1 ^d-g, AB^
TC-CPDP	21.2 ± 0.2 ^b, A^	27.8 ± 0.6 ^fg, B^	2.4 ± 0.1 ^l, C^	2.4 ± 0.3 ^l, C^
TC-T	30.5 ± 0.2 ^hij, C^	33.1 ± 0.8 ^l, DE^	1.1 ± 0.1 ^cde, A^	1.4 ± 0.1 ^e-h, B^
**Savoy**				
SC-HSC	24.5 ± 1.0 ^e, A^	24.6 ± 1.43 ^e, A^	3.7 ± 0.1 ^m, A^	4.7 ± 0.3 ^n, B^
SC-PW	24.1 ± 0.1 ^cde, A^	24.3 ± 0.3 ^de, A^	5.3 ± 0.1 ^o, BC^	6.4 ± 0.5 ^q, D^
SC-SDG	dng	24.4 ± 0.5 ^de, A^	dng	5.8 ± 0.7 ^p, CD^
**Red**				
RC-RL	21.0 ± 0.5 ^b, A^	33.6 ± 0.6^l, C^	1.6 ± 0.1 ^ghi, B^	0.9 ± 0.1 ^a-d, A^
RC-RM	dng	35.4 ± 1.0 ^m, D^	dng	1.5 ± 0.3 ^f-i, B^
RC-RD	25.3 ± 0.1 ^e, B^	36.7 ± 0.7 ^m, E^	2.1 ± 0.0 ^jkl, C^	1.9 ± 0.1 ^ijk, C^
**White**				
WC-FEM	21.2 ± 0.9 ^b, A^	21.3 ± 0.4 ^b, A^	3.8 ± 0.2 ^m, C^	3.4 ± 0.2 ^m, B^
WC-CRB	22.8 ± 0.6 ^c, B^	23.0 ± 1.2 ^cd, B^	2.2 ± 0.2 ^kl, A^	2.1 ± 0.1 ^kl, A^

Values are means of three processing replicates and two technical replicates (*n* = 6 ± SD). SD: standard deviation from mean; dng: did not grow. Letters “A-E”: mean values not sharing a common uppercase letter differ significantly (*p* < 0.05) between accessions and growing condition within a cabbage type for each parameter (i.e., protein content and specific activity). Letters “a-q”: mean values not sharing a common lowercase letter differ significantly (*p* < 0.05) between cabbage types, accessions, and growing condition for each parameter (i.e., protein content and specific activity). See Table 1 for full names of cabbage accessions.

## Data Availability

The data presented in this study are available on request from the corresponding author.

## Supplementary Materials

The following are available online at https://www.mdpi.com/article/10.3390/foods10122903/s1, Figure S1: Cross-section of planted cabbage morphotypes (**a**) Black kale (**b**) Wild cabbage (**c**) Tronchuda cabbage (**d**) Savoy cabbage (Field grown) (**e**) Savoy cabbage (Glasshouse grown) (**f**) Red cabbage (Field grown) (**g**) Red cabbage (Glasshouse grown) (**h**) White cabbage (Field grown) (**i**) White cabbage (Glasshouse grown). Figure S2: Cross-section of cabbages grown under (**a**) Controlled environment and (**b**) Glasshouse. Figure S3: Examples of GC-MS chromatograms for field and glasshouse grown samples for each morphotype of cabbage studied (**a**) Black kale; (**b**) Wild cabbage; (**c**) Tronchuda cabbage; (**d**) Savoy cabbage; (**e**) Red cabbage; and (**f**) White cabbage. Table S1: Climatic data of field and glasshouse cabbages. Table S2: Glucosinolate concentration in cabbages grown under different conditions (mg/g DW). Table S3: Glucosinolate hydrolysis products concentration in cabbages grown under different conditions (µg/g DW sulforaphane equivalent). Table S4: Pearson correlation matrix table showing correlations between glucosinolates and glucosinolate hydrolysis products identified in cabbage grown under two different conditions (a) correlation coefficients (r) and (b) significance of the correlation (p value).
