# Supplementary material for: Influence of Cabbage (Brassica oleracea) Accession and Growing Conditions on Myrosinase Activity, Glucosinolates and Their Hydrolysis Products
Source: Foods. 2021 Nov 23;10(12):2903. doi: 10.3390/foods10122903 (PMC8700869; doi:10.3390/foods10122903)
Supplement: Supplementary file 1 [file foods-10-02903-s001.zip › Supplementary Figures_paper 1.pdf]

Supplementary Figures

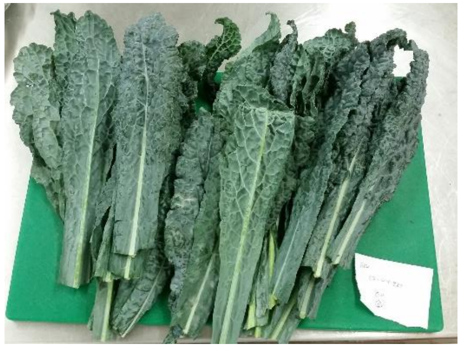

(**a**) Black kale

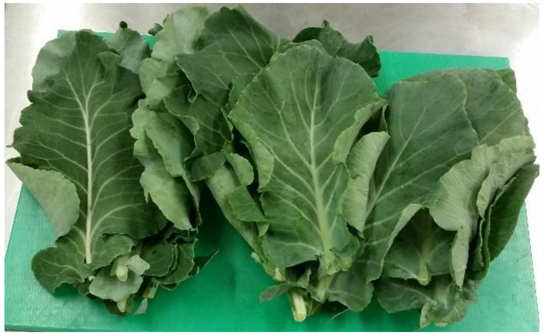

(**b**) Wild cabbage

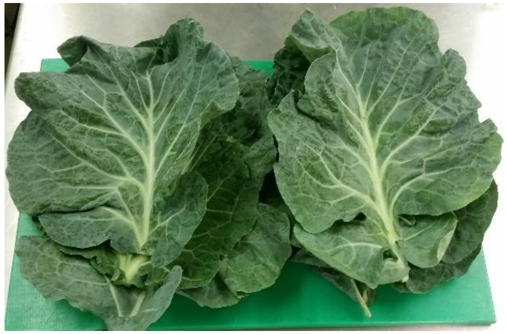

(**c**) Tronchuda cabbage

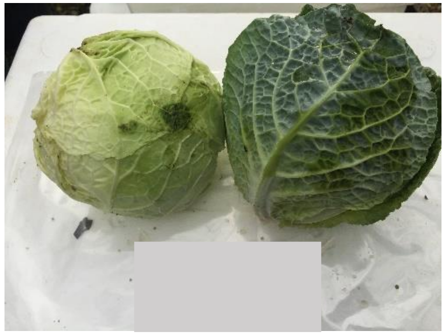

(**d**) Savoy cabbage (Field)

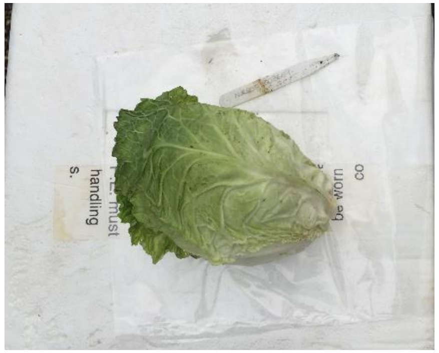

(**e**) Savoy cabbage (Glasshouse)

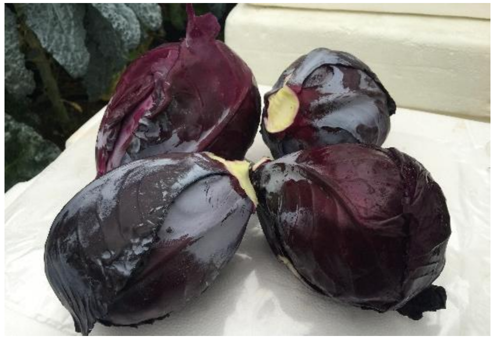

(**f**) Red cabbage (Field)

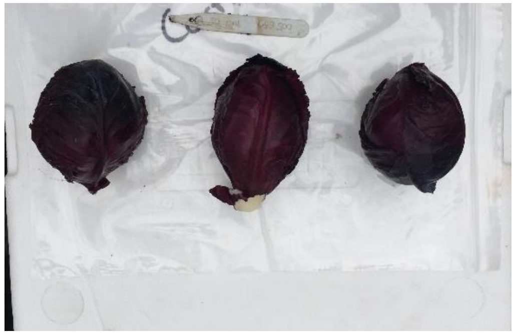

(**g**) Red cabbage (Glasshouse)

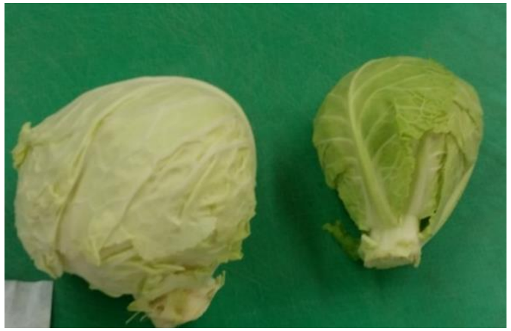

(**h**) White cabbage (Field)

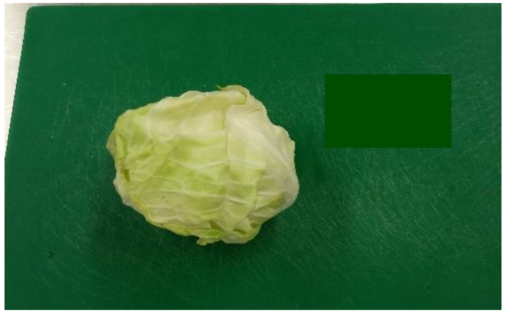

(**i**) White cabbage (Glasshouse)

Figure S1. Cross-section of planted cabbage morphotypes (**a**) Black kale (**b**) Wild cabbage (**c**) Tronchuda cabbage (**d**) Savoy cabbage (Field grown) (**e**) Savoy cabbage (Glasshouse grown) (**f**) Red cabbage (Field grown) (**g**) Red cabbage (Glasshouse grown) (**h**) White cabbage (Field grown) (**i**) White cabbage (Glasshouse grown).

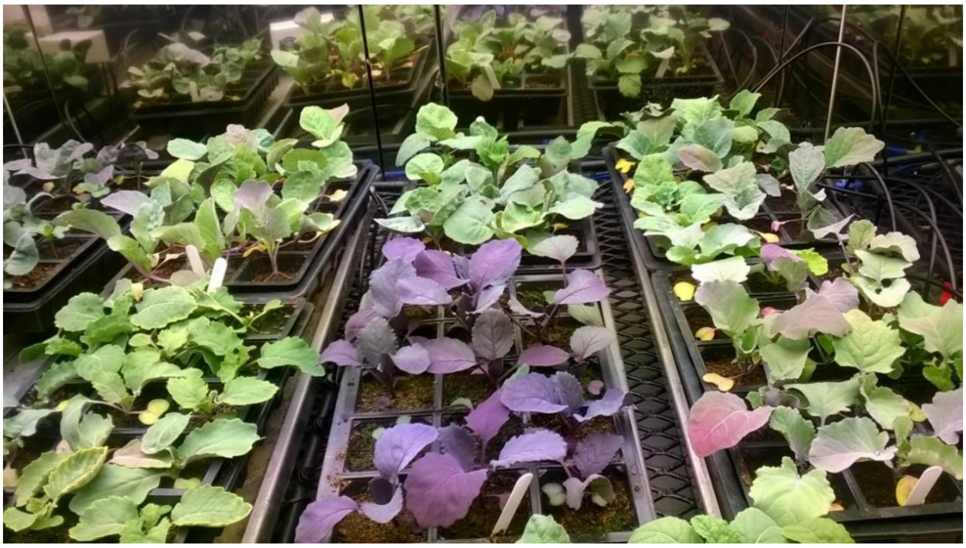

(**a**) Controlled environment

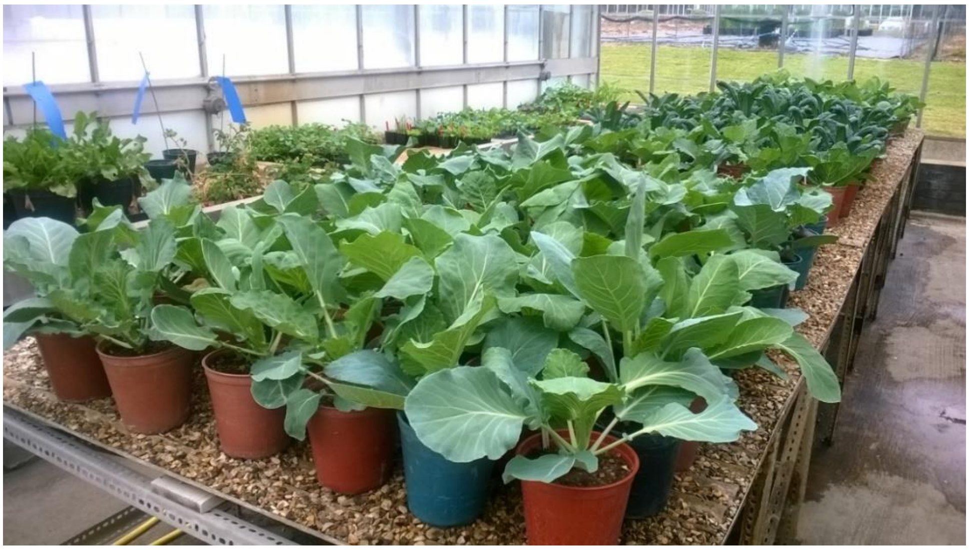

(**b**) Glasshouse

Figure S2. Cross-section of cabbages grown under (**a**) Controlled environment and (**b**) Glasshouse.

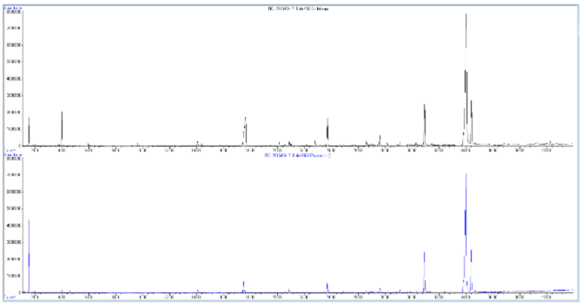

(**a**) Black kale

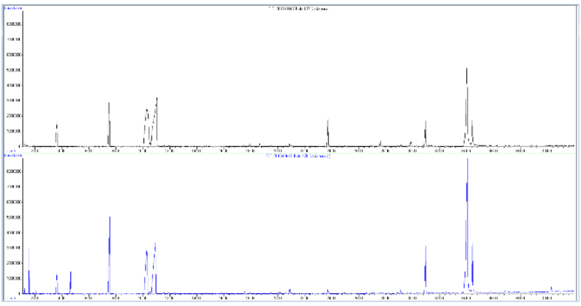

(**b**) Wild cabbage

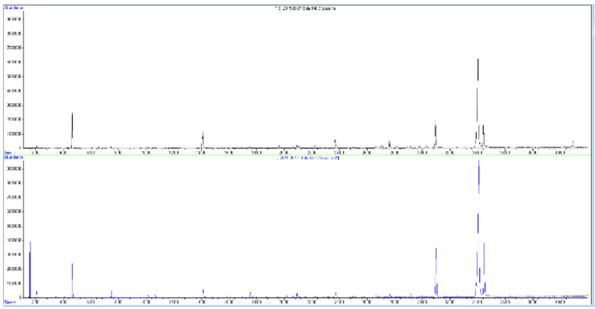

(**c**) Tronchuda cabbage

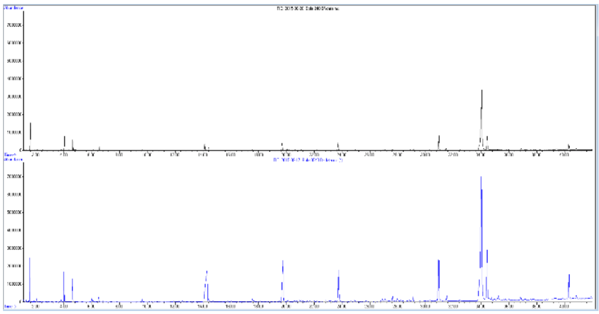

(**d**) Savoy cabbage

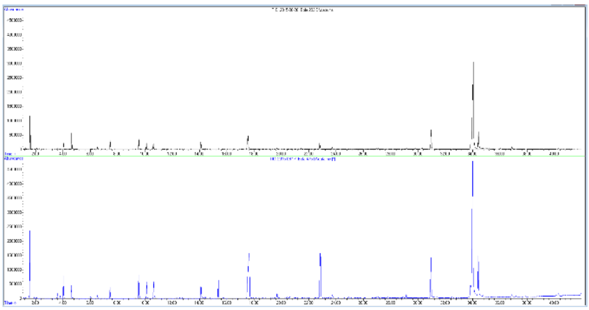

(**e**) Red cabbage

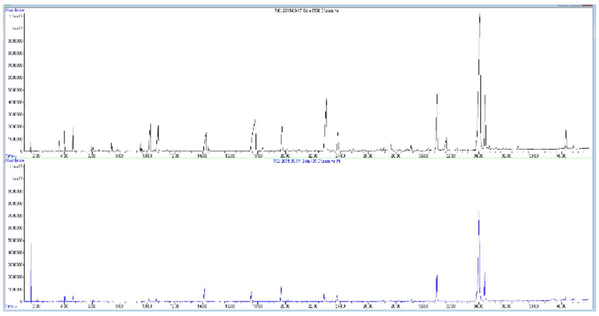

(**f**) White cabbage

Figure S3. Examples of GC-MS chromatograms for field and glasshouse grown samples for each morphotype of cabbage studied (**a**) Black kale; (**b**) Wild cabbage; (**c**) Tronchuda cabbage; (**d**) Savoy cabbage; (**e**) Red cabbage and (**f**) White cabbage. Black coloured chromatograms represent field grown samples while blue coloured chromatograms are glasshouse grown samples for each cabbage morphotype.
